# Identifying Tampered Radio-Frequency Transmissions in LoRa Networks Using Machine Learning

**DOI:** 10.3390/s24206611

**Published:** 2024-10-14

**Authors:** Nurettin Selcuk Senol, Amar Rasheed, Mohamed Baza, Maazen Alsabaan

**Affiliations:** 1 Department of Computer Science, Sam Houston State University, Huntsville, TX 77340, USA; nss016@shsu.edu (N.S.S.); axr249@shsu.edu (A.R.); 2 Department of Computer Science, College of Charleston, Charleston, SC 29424, USA; 3 Department of Computer Engineering, College of Computer and Information Sciences, King Saud University, P.O. Box 51178, Riyadh 11543, Saudi Arabia; malsabaan@ksu.edu.sa

**Keywords:** anomaly detection, LoRa, cybersecurity, IoT, frequency analysis, machine learning

## Abstract

Long-range networks, renowned for their long-range, low-power communication capabilities, form the backbone of many Internet of Things systems, enabling efficient and reliable data transmission. However, detecting tampered frequency signals poses a considerable challenge due to the vulnerability of LoRa devices to radio-frequency interference and signal manipulation, which can undermine both data integrity and security. This paper presents an innovative method for identifying tampered radio frequency transmissions by employing five sophisticated anomaly detection algorithms—Local Outlier Factor, Isolation Forest, Variational Autoencoder, traditional Autoencoder, and Principal Component Analysis within the framework of a LoRa-based Internet of Things network structure. The novelty of this work lies in applying image-based tampered frequency techniques with these algorithms, offering a new perspective on securing LoRa transmissions. We generated a dataset of over 26,000 images derived from real-world experiments with both normal and manipulated frequency signals by splitting video recordings of LoRa transmissions into frames to thoroughly assess the performance of each algorithm. Our results demonstrate that Local Outlier Factor achieved the highest accuracy of 97.78%, followed by Variational Autoencoder, traditional Autoencoder and Principal Component Analysis at 97.27%, and Isolation Forest at 84.49%. These findings highlight the effectiveness of these methods in detecting tampered frequencies, underscoring their potential for enhancing the reliability and security of LoRa networks.

## 1. Introduction

**Figure 1:**
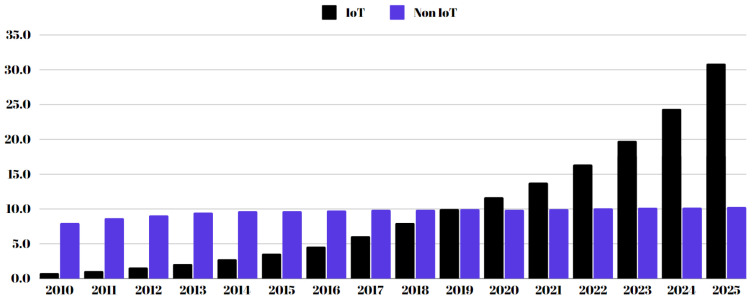
The number of active connections for both Internet of Things and non-IoT devices worldwide from 2010 to 2025 is measured in billions [3].

LoRa (LoRa) networks provide reliable, low-power, long-range wireless communication capabilities, making them a crucial component of the Internet of Things (IoT) ecosystem. This technology is ideal for applications like smart cities, environmental monitoring, precision agriculture, and industrial IoT systems because it facilitates low-data-rate communication between battery-operated sensors dispersed over large areas. As shown in Figure 1, the number of active IoT connections worldwide is projected to grow significantly compared to non-IoT connections from 2010 to 2025, reflecting the increasing adoption of IoT technologies in such domains. The rapid adoption of LoRa has been fueled by its low power consumption, which enables devices to work for years on a single battery, and its ability to maintain communication over distances of up to 10 km in rural areas. Industry reports state that the growing demand for IoT solutions across multiple sectors is expected to propel the global LoRa market towards considerable expansion [1,2].

However, LoRa networks also bring critical challenges, particularly from a cybersecurity and digital forensics perspective. IoT devices handle sensitive data and often have vulnerabilities that make them attractive targets for cyberattacks. Ensuring the security of IoT networks, including LoRa-based networks, is vital to protect data integrity and service reliability. Tampered radio frequencies in LoRa-based networks can open the door for attackers to launch various forms of attacks, such as jamming or signal spoofing, which can degrade network performance or cause complete service disruption. Attackers can intercept and manipulate frequency signals to inject false data, delay transmissions, or alter packet contents, ultimately compromising both data integrity and confidentiality. Furthermore, such vulnerabilities can be exploited to eavesdrop on sensitive information, manipulate control systems, or disrupt critical IoT applications, leading to cascading effects on operational processes and user safety.

In the context of LoRa-based networks, the effectiveness of the proposed approach could be significantly influenced by various types of attacks, such as jamming, signal spoofing, or replay attacks. While the approach may perform well under normal conditions, it is important to assess its resilience when subjected to these attack vectors. Jamming attacks, for instance, can overwhelm the network by flooding the frequency spectrum, leading to packet loss or delays that the current method may not handle efficiently. Signal spoofing can inject falsified data into the network, potentially misleading the detection system into categorizing anomalies incorrectly. Similarly, replay attacks, where previously transmitted signals are captured and retransmitted, could compromise the approach’s ability to differentiate between legitimate and manipulated signals. Without evaluating these attack scenarios, it is difficult to fully understand how robust and reliable the proposed method is in real-world applications where attackers may exploit such vulnerabilities. Adding this assessment would strengthen the scientific rigor and practical applicability of the approach.

This paper focuses on addressing the challenge of detecting tampered LoRa frequencies, an essential task for ensuring the integrity of LoRa-based IoT systems. We propose a machine learning-based approach that leverages image-based anomaly detection techniques to identify deviations in LoRa frequency patterns. Our novel contribution lies in the application of advanced algorithms, such as Autoencoder, Local Outlier Factor, Principal Component Analysis, Variational Autoencoder, and Isolation Forest, specifically tailored for the LoRa ecosystem.

This work presents a novel approach to anomaly detection in LoRa-based IoT systems by the combination of multiple machine learning techniques and their application to a newly generated dataset specifically designed for this work. This work is motivated by the rising reliance on LoRa-based communication networks and the quick proliferation of IoT devices, which call for robust and efficient anomaly detection techniques to protect system security and integrity. When handling the unique characteristics of IoT data, such as its high dimensionality, diversity, and temporal fluctuations, conventional anomaly detection algorithms frequently fail. We conducted real-world experiments to create a unique dataset that included both modified and standard frequency signals in order to address these issues. This methodology enables a more accurate and comprehensive evaluation of our integrated system.

Our approach is well-suited for large-scale IoT environments and provides domain-specific insights into the unique challenges of anomaly detection in LoRa networks. Additionally, by employing image-based data for anomaly detection, this work offers a novel perspective, further enhancing detection capabilities and supporting the security and performance of IoT systems. The decision to use image-based data for anomaly detection in LoRa networks stems from the need to capture and visualize complex patterns within frequency signals that might not be apparent through traditional data formats. By converting frequency signal characteristics into images, subtle anomalies, such as interference or tampering, become more discernible, allowing for more effective detection. This method utilizes advanced algorithms commonly employed in image processing and computer vision, including Principal Component Analysis, Autoencoders, and Isolation Forests. These algorithms are adept at detecting spatial patterns and anomalies from expected behavior, providing a sophisticated level of security to identify tampered transmissions and protect IoT networks from malicious threats. The contribution of this paper is as follows:

- We have generated a comprehensive dataset consisting of benign samples and tampered signals on LoRa transmissions. The dataset captures both normal and manipulated frequency signals, offering a diverse collection of real-world data for thorough analysis.
- We provide an anomaly detection system for LoRa networks that is based on machine learning. To identify tampered radio frequencies in LoRa-based IoT systems, our method combines several sophisticated algorithms, including Autoencoder, Local Outlier Factor (LOF), Principal Component Analysis (PCA), Variational Autoencoder, and Isolation Forest. Several challenges have been addressed in applying these algorithms to identity tampered signals on LoRa transmissions.
- Using real datasets, extensive experiments have been conducted, revealing significant improvements in detection performance. The Local Outlier Factor (LOF) algorithm achieved the highest detection accuracy at 97.78%, closely followed by the Variational Autoencoder, Principal Component Analysis, and Autoencoder with 97.27% accuracy, respectively, while the Isolation Forest demonstrated lower performance at 84.49%.

This is how the remainder of the article is structured: A thorough assessment of prior research on anomaly detection in LoRa networks is provided in Section 2. The hardware requirements and system architecture are explained in Section 3. Section 4 describes the methodology used in our research, including how machine learning techniques were applied and how data were collected. The tests carried out and the outcomes discussed are covered in Section 5. Lastly, Section 6 summarizes the findings, discusses the work’s shortcomings, and makes recommendations for possible future research topics.

## 2. Related Works

This section of our work provides a comprehensive overview of existing research related to IoT devices and LoRa technology. It critically examines previous studies, highlighting the advancements and gaps in the current body of knowledge. By exploring various scholarly articles, technical reports, and case studies, this section aims to establish a solid foundation for understanding the context and significance of our investigation. Additionally, it identifies the key challenges and emerging trends in the field, setting the stage for the subsequent sections of this article. This review underscores the revolutionary impact of IoT in various sectors, the crucial role of LoRa technology in enabling long-range communication, and the importance of robust digital forensic techniques to ensure cybersecurity in the ever-expanding IoT ecosystem.

In order to guarantee the dependability and security of Internet of Things networks that use LoRa technology—a long-range, low-power wireless communication protocol—anomaly detection is essential. Anomaly detection systems can detect issues like interference, jamming, or unauthorized access by spotting departures from typical operational patterns, such as irregular frequency utilization or strange data transmission behaviors. Babazadeh et al. [4] focused on developing an edge-based anomaly detection system for LoRa-based wireless sensor networks. Similarly, in [5], a water quality monitoring system (IoT-WQMS) prototype was developed by researchers by combining an anomaly detection algorithm, a LoRa repeater, and IoT technologies. This system effectively extends the range of LoRa communication, reduces packet loss, and improves anomaly detection accuracy, thereby enhancing the reliability of real-time water quality monitoring.

In reference [6], researchers developed an innovative distributed data analytics framework for detecting anomalies in smart water networks. Their approach involved high-speed edge analytics using a single-core Intel Curie processor and a tailored Lempel-Ziv compression algorithm. This method allowed them to transmit only the compressed anomaly-related data to the server via LoRa. The effectiveness of this approach was experimentally validated, demonstrating its high resource efficiency.

Kurniawan et al. [7] developed an anomaly detection system to enhance the security of LoRaWAN gateway devices. They employed machine learning models and various outlier detection algorithms to analyze incoming packet data, and they developed a specialized dataset from LoRaWAN gateways. Their research demonstrated that machine learning is a viable and accurate method for detecting anomalies in constrained LoRaWAN devices. This approach differs from our work, which utilizes a distinct methodology and dataset creation strategy for LoRa networks.

Although Michael Mesarcik et al. [8] did not focus on LoRa signals, their research methodology bears similarities to our own work. They developed an anomaly detection framework for radio telescopes, utilizing a dataset of 7,050 autocorrelation-based spectrograms from the LOFAR telescope, each labeled with 10 different types of anomalies. Their framework, the Radio Observatory Anomaly Detector (ROAD), integrates Self-Supervised Learning (SSL) with supervised classification to identify both common and rare anomalies. ROAD is capable of processing each spectrogram in under 1 millisecond and achieves an F-2 score of 0.92 for overall anomaly detection, along with a 2% false positive rate. It also attained an F-2 score of 0.89 for per-class classification, demonstrating superior performance compared to similar methods.

CNNs have become an effective technique for anomaly identification based on images. For example, a simple method using CNNs to categorize malware photos in Internet of Things environments has demonstrated encouraging outcomes [9]. Moreover, an integrated model that leverages the sequential data properties present in Internet of Things applications to detect anomalies has been suggested [10]. This model combines CNNs with recurrent autoencoders.

A binary anomaly discriminator and a pre-trained feature extractor are also used by SimpleNet, a network intended for anomaly detection and localization. The significance of feature extraction in enhancing detection accuracy is emphasized by this technique [11]. The possibilities for real-time anomaly detection in resource-constrained IoT contexts are highlighted by the developments in CNN architectures.

Enhancing anomaly detection skills has been made possible by the incorporation of computer vision techniques. One noteworthy technique is the conversion of time series signals into image vectors, which makes deep learning techniques easier to use for anomaly classification. Ref. [12] demonstrated the effectiveness of this approach in structural health monitoring, highlighting the versatility of picture representation in anomaly detection applications.

A transformer-based framework for multivariate time-series anomaly detection is one of the most recent innovations. This approach offers a reliable method for identifying anomalies in Internet of Things environments by learning a graph structure and capturing temporal dependencies [13]. These developments show a move toward more complicated architectures that can understand intricate data linkages.

LOF, Autoencoder, and Isolation Forest are well-known methods in anomaly identification. We examine how these methods have been used with different anomaly detection datasets in this section of our article. As an example, a Convolutional Recurrent AutoEncoder (CR-AE) was created in the work of Wang et al. [14] to identify anomalies in video data. This model enhances feature extraction through attention processes by combining a convolutional autoencoder with an attention-based Convolutional Long-Short-Term Memory (ConvLSTM) network to capture both temporal and spatial anomalies. The efficacy of the CR-AE in detecting anomalies in video sequences was assessed using the UCSD ped2 and Avenue datasets, yielding frame-level AUC ratings of 95.6% and 73.1%, respectively. Nonetheless, the intricate architecture that merges attention-based ConvLSTM networks with Convolutional AutoEncoders results in high computational demands, posing challenges for real-time applications, particularly in environments with limited resources.

In reference [15], an innovative method for detecting anomalies in High-Performance Computing (HPC) systems was introduced, utilizing autoencoders, a type of deep learning neural network. Unlike prior approaches that required learning about anomalies from small datasets, this strategy involves training autoencoders to learn the usual operational patterns of supercomputer nodes and then discover departures from this norm. The method was evaluated on a real supercomputer with a comprehensive monitoring setup and achieved high accuracy in detecting previously unseen anomalies, with performance ranging from 88% to 96%. However, like many deep learning models, this method faces a challenge in interpretability, making it difficult to understand the reasons behind flagged anomalies, which can be a critical issue in HPC operations where clear explanations are essential for effective diagnosis and remediation.

Choi et al. [16] introduced an innovative anomaly detection framework named HFR-AE, which projects new data into a subspace where the autoencoder achieves high-fidelity reconstruction. Comparing this method to traditional Autoencoder-based techniques, the area under the receiver operating characteristic curve (AUROC) increases by as much as 13.4%, indicating an improvement in the ability to distinguish between normal and anomalous signals. However, the need to project inputs into this high-fidelity subspace introduces greater computational complexity, which may pose challenges for scalability in real-time applications or resource-limited environments, such as edge IoT devices.

The authors of [17] conducted a literature review on local outlier detection algorithms, focusing particularly on the Local Outlier Factor and its application to both static datasets and data streams. This review is relevant to our research as it provides a comprehensive analysis of the strengths and weaknesses of existing algorithms, helping to inform the selection of the Local Outlier Factor for our anomaly detection approach. Additionally, the proposed improvements for handling high-velocity data streams align with the challenges we face in real-time LoRa network monitoring, making their findings directly applicable to enhancing our system’s detection capabilities. As a practical example, the work in [18] developed a novel hyperspectral anomaly detection method that integrates low-rank representation (LRR) with an LOF-based adaptive filter. This approach improves detection performance by refining the dictionary of background pixels and enhancing the differentiation between anomalies and background using intrinsic spatial structure, outperforming several existing methods on real-world datasets. However, its reliance on a refined dictionary of background pixels may increase the computational burden, potentially limiting its applicability in scenarios with large-scale or rapidly changing data streams.

Xu et al. [19] proposed an innovative heuristic approach for optimizing hyperparameters in the Local Outlier Factor (LOF) model, aimed at tackling the issue of detecting previously unseen anomalies in IoT systems. This work is directly relevant to our research, as optimizing hyperparameters is crucial for improving the accuracy of LOF in anomaly detection. Their findings offer valuable insights into enhancing the model’s predictive performance, which we apply in our study to improve the detection of anomalies in LoRa-based IoT networks, ensuring better adaptability to real-world scenarios. Additionally, Hendrycks et al. [20] introduced a technique known as Outlier Exposure (OE) to improve deep anomaly detection. This technique involves training models with an additional dataset of outliers to enhance their ability to generalize and identify novel anomalies. Their extensive research indicated that OE significantly enhances detection performance and addresses challenges where generative models might incorrectly assign high probabilities to outlier images. However, the dependence on heuristic techniques in these approaches may restrict their ability to generalize across highly varied datasets and contexts.

A dynamic adaptive local outlier factor (DALOF) technique was presented in [21] and is intended to identify anomalous data segments in rolling bearings in large-scale industrial settings. This method includes a data quality evaluation model, a dynamic segmentation process, and Principal Component Analysis (PCA) for time-domain feature extraction. It has shown enhanced accuracy in detecting anomalies compared to traditional methods. However, the dynamic segmentation and PCA processes may introduce significant computational overhead, which could affect real-time performance. Additionally, the method’s dependence on precise parameter tuning for optimal results and its potential difficulties in adapting to different domains without modifications pose further challenges. Moreover, the method’s reliance on high-quality data means that poor or noisy inputs could reduce its effectiveness.

The authors of [22] proposed an innovative two-stream Isolation Forest (IF) approach for hyperspectral anomaly detection (HAD), which enhances anomaly sensitivity by integrating deep spectral features with spatial information. This method employs both global and local weighted Isolation Forests, incorporates morphological and Gaussian filters for spatial detection, and demonstrates superior performance compared to current leading techniques across five hyperspectral datasets.

In conclusion, this literature review highlights the significant advancements and ongoing challenges in anomaly detection across various domains, including IoT systems, hyperspectral imaging, and high-performance computing. The reviewed studies reveal a trend toward integrating advanced techniques, such as deep learning models, heuristic methodologies, and sophisticated feature extraction methods, to enhance detection accuracy and efficiency. Notably, the incorporation of novel frameworks like autoencoders, Isolation Forest, and LOF demonstrates promising improvements in handling unseen anomalies and refining detection models. However, gaps remain in fully exploiting spectral-spatial information, which combines data across different wavelengths with spatial context and is crucial for accurate anomaly detection in fields like hyperspectral imaging. For instance, existing models frequently struggle to incorporate spatial patterns effectively in real-time IoT systems or rapidly changing environments, which restricts their performance in high-speed data contexts. Future studies should work to overcome these constraints by developing more adaptable and robust anomaly detection techniques that make use of both spectral and spatial data and by carefully evaluating their performance in a variety of real-world scenarios.

## 3. System Architecture

In this section, we describe the hardware utilized to construct the LoRa testbed, which serves as the foundational system for this research. The detailed steps involved in developing this testbed are outlined in [23].

### Hardware Components

The proposed system architecture for anomaly detection in LoRa networks incorporates two **MKRWAN1310** devices and two **HackRF** devices, each contributing critical capabilities for monitoring and analyzing LoRa transmissions.

**Figure 2:**
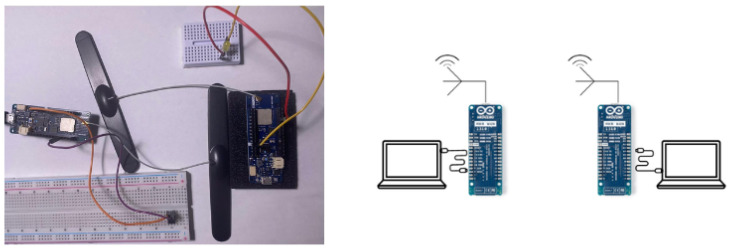
Experimental setup for data collection using HackRF for frequency manipulation. The system consists of two MKRWAN 1310 devices for wireless signal transmission and reception, interfaced with computers for monitoring and control [23].

**Figure 3:**
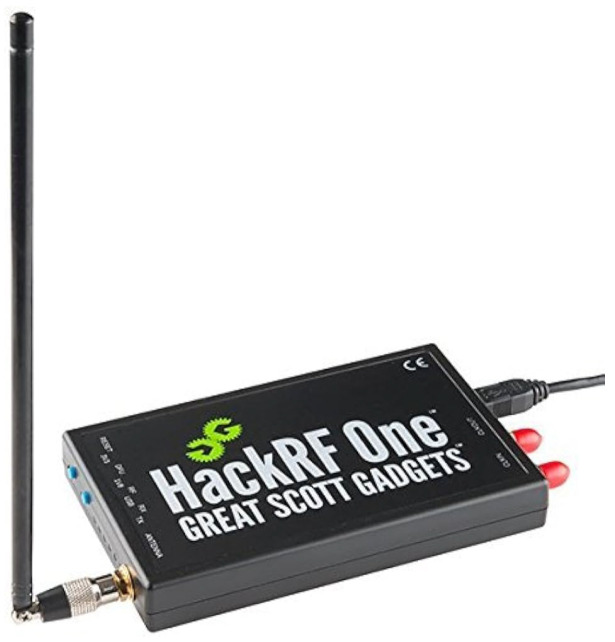
HackRf have been utilized for manipulation of the system in Figure 2. A software-defined radio (SDR) device, the HackRF, can send and receive radio signals in the 1 MHz to 6 GHz frequency range.

1. The **MKRWAN1310** devices serve as both the transmitter and receiver units in the LoRa network, playing a central role in data collection and anomaly detection. Figure 2 demonstrates the setup that we use. Each MKRWAN1310 device operates at a frequency of 915 MHz with a bandwidth of 500 kHz and a spreading factor of 5, ensuring effective and reliable communication within the LoRa network. The devices are equipped with an Atmel ATSAMD21 microcontroller featuring a 32-bit ARM Cortex-M0+ core, 256 KB of flash memory, and 32 KB of SRAM. They offer various interfaces, including UART, SPI, and I2C, and operate within a voltage range of 3.3 V to 5 V. Integrated with the Semtech SX1276 LoRa transceiver, the MKRWAN1310 supports long-range communication with an adaptive data rate and low power consumption.
2. The **HackRF** devices are employed to capture and analyze the radio frequency spectrum of the LoRa transmissions. Each HackRF device covers a wide frequency range from 1 MHz to 6 GHz and supports a maximum sample rate of 20 MS/s with an 8-bit ADC resolution. The devices feature a maximum instantaneous bandwidth of 10 MHz, are connected via USB 2.0, and are powered by a 5 V supply. The direct conversion architecture of the HackRF, combined with its broad frequency range, enables detailed signal analysis and effective detection of anomalies. Figure 3 shows the HackRF device that we use utilized in this work.

Despite their advanced capabilities, HackRF devices are susceptible to signal interference from external RF sources, electronic devices, and environmental factors. To address this, the system employs shielding and isolation techniques, alongside careful calibration. Optimal frequency ranges and high-quality filters are also used to reduce the impact of unwanted signals, ensuring accurate and reliable detection of anomalies in LoRa communications. To enhance the performance of HackRF devices and reduce signal interference, the system employs the following key techniques:

- **Shielding and Isolation:** HackRF devices are enclosed in conductive materials to block external RF signals and minimize ambient noise from nearby electronics.
- **Calibration:** A meticulous calibration process adjusts parameters like gain and frequency offsets to align the device with the expected signal characteristics, ensuring accurate reception and transmission.
- **Frequency Optimization:** The system selects optimal frequency ranges to reduce interference from other wireless systems, focusing on the LoRa signals of interest.
- **High-Quality Filters:** Bandpass filters are used to allow only the desired frequency range to pass, effectively isolating LoRa signals from unwanted background noise.

These techniques collectively ensure reliable monitoring and precise anomaly detection within LoRa networks.

This architecture effectively integrates the MKRWAN1310 and HackRF devices, enabling comprehensive monitoring and precise anomaly detection within LoRa networks.

HackRF is a flexible software-defined radio (SDR) platform capable of both transmitting and receiving radio signals across a broad frequency spectrum. When applied to LoRa frequency manipulation, HackRF can be set up to engage with multiple LoRa signals [24].

To use HackRF with LoRa, we begin by installing the necessary drivers and software to interface with HackRF. We utilized AirSpy SDR [25] for frequency monitoring. Next, HackRF is configured to operate within the frequency range used by LoRa devices, typically in the ISM bands; for our case, 915 MHz is used in North America.

By employing appropriate software tools, such as GNU Radio (version number 3.10.8.0) or SDR (AirSPY v1.0.0.1919), HackRF (v. 2024.02.1) can effectively monitor LoRa signals, enabling users to detect anomalies or interference within the frequency spectrum. Additionally, HackRF can generate signals within the LoRa frequency bands, making it useful for creating test signals or simulating network interference. However, working with LoRa signals requires specific software libraries that support LoRa modulation to decode or encode LoRa packets. While HackRF is capable of tuning to LoRa frequencies and generating signals, more advanced manipulation of LoRa communications, such as message injection or jamming, demands a deeper understanding of LoRa’s modulation scheme and may necessitate custom software solutions. In our scenario, a HACK RF device is employed to execute a kind of Man-in-the-Middle (MitM) attack by intercepting and manipulating the radio frequency signals between an MKRWAN 1310 transmitter and an MKRWAN 1310 receiver. This manipulation of the signals resulted in some packets being observed as not being delivered correctly. Experiment details will be in our experiment and results section.

## 4. Proposed Methodology

**Figure 4:**
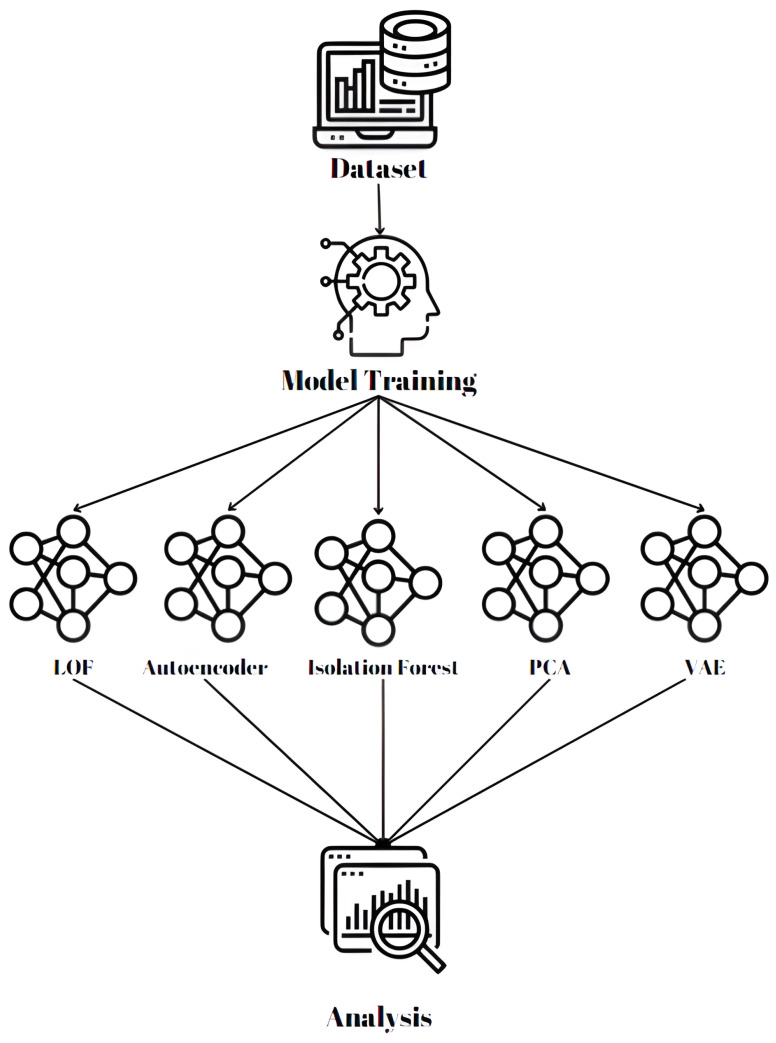
Flowchart depicting the methodology for image-based anomaly detection using multiple machine learning models.

The methodological approaches used in this work to design and assess frequency image detection and classification on embedded systems are shown in Figure 4. It illustrates the methodology for detecting anomalies in LoRa networks using multiple machine learning models. It begins with a dataset, which undergoes model training using various algorithms such as LOF, Autoencoder, Isolation Forest, Principal Component Analysis, and Variational Autoencoder. The results from these models are then analyzed to determine the best-performing approach for identifying anomalies in the LoRa broadcast environment. The steps in our methodology are:

1. Gathering of Dataset and Preparation: The work starts by gathering a large dataset of frequency pictures produced by radio-frequency broadcasts in an IoT network based on LoRa. Both regular and anomalous transmissions are included in the dataset, which is then used to train and assess the models. This dataset includes both normal and anomalous transmissions, providing a diverse representation of signal behaviors crucial for training and evaluating the models. Following the gathering process, several data preparation steps were undertaken to ensure the dataset’s integrity and effectiveness for machine learning applications. First, each image in the dataset was resized to a standardized dimension to maintain consistency across the data. This resizing facilitated efficient processing and ensured that all input data conformed to the expected dimensions for the machine learning models. Next, pixel normalization was performed, scaling the pixel values to a range of [0, 1]. This normalization is essential as it enhances the convergence of the training algorithms, allowing the models to learn effectively from the data. Moreover, the dataset was carefully labeled, distinguishing between normal and anomalous signals based on their characteristics. This labeling not only supported supervised learning techniques but also enabled a structured approach for analyzing the performance of different anomaly detection algorithms. To further enrich the dataset, data augmentation techniques were applied. These included transformations such as rotation, flipping, and the addition of random noise, which helped to increase the dataset’s diversity and robustness. By simulating variations in the signal data, these augmentations allowed the models to generalize better and improve their ability to detect anomalies in real-world applications.
2. A variety of machine learning models are utilized in the anomaly detection process. To identify any deviations from the norm and learn the features of typical data, each of these models is trained independently on the dataset (anomalies). Figure 4 shows a flowchart for the methodology of image-based anomaly detection. The models that were used in this research are:
  - **Local Outlier Factor (LOF)**: A density-based technique that analyzes a data point’s local density with respect to its neighbors to find anomalies.
  - **Autoencoder**: A neural network model that has been taught to recreate the source data. Any notable inaccuracy in reconstruction points to an oddity.
  - **Isolation Forest**: A tree-based method that effectively identifies outliers by separating values and arbitrarily choosing attributes to isolate data.
  - **Principal Component Analysis (PCA)**: A method of reducing the dimensionality of data by breaking down large, multidimensional data into a smaller group of uncorrelated elements known as principle components that together account for the majority of the variance in the data. By keeping the key patterns and traits, it helps streamline datasets and make them simpler to analyze.
  - **Variational Autoencoder**: A Variational Autoencoder is a generative model that combines neural networks with probabilistic graphical models, allowing it to learn a latent representation of input data while generating new data samples from that representation.
3. Evaluation: The models are used to evaluate the dataset after they have been trained. The outputs produced by each model show whether a transmission is abnormal or not. Next, these models’ performance is assessed using criteria, including precision, recall, and total accuracy. The best model for identifying anomalies in LoRa broadcasts is determined in part by this evaluation.

The proposed Algorithm 1 describes an approach for implementing a model-based anomaly detection. The pseudocode begins by outlining the process of loading, preprocessing, and splitting the dataset, followed by training multiple models using cross-validation, and finally identifying anomalies in the test set. This method works well for image-based anomaly detection since it combines the strength of several models to improve model performance. The framework can reliably identify variations in the dataset that are suggestive of anomalies since it is trained using numerous models with varying configurations and uses reconstruction error as a measure for anomaly detection.
**Algorithm 1** Pseudocode for Multi-Model Anomaly Detection 1:D←LoadData()                                          ▹ Load dataset from predefined directories 2:**if** any $d_{k}\in D$ is missing or invalid, apply imputation 3:Convert *D* into a feature matrix: $X=[x_{1},x_{2},\ldots ,x_{n}]$ 4:Apply normalization to *X*: X=normalize(X) 5:Split *X* into training set $X_{train}$ and validation set $X_{val}$ 6:models←[IsolationForest,VariationalAutoencoder,…]           ▹ Array of candidate models 7:predictions←[0]×len(models)                                         ▹ Array for model predictions 8:runs←10 9:avg_prediction←010:**for** each model in models **do**11:    **for** i←1 to runs **do**12:        Initialize the current model13:        Fit the model using $X_{train}$14:        current_prediction← Evaluate the model on $X_{val}$15:        avg_prediction +=current_prediction16:    **end for**17:    predictions[model]←avg_prediction/runs18:    Display evaluation metrics for the current model19:    Reset avg_prediction←020:**end for**

### 4.1. Dataset

For the dataset creation in this study, we leveraged our previously developed system as detailed in [23], along with specialized equipment including the AirSPY SDR and HACKRF devices to monitor normal frequency transmissions in a LoRa-based IoT setup. The experiment was conducted over a continuous four-hour period, during which we ensured successful packet reception by the receiver while monitoring the process in real-time using Software-Defined Radio (SDR) tools.

The Windows video recording feature was utilized to capture the live transmission frequencies. Once the transmission process was completed, we extracted individual frames from the recorded video using a Python script. This step allowed us to convert the visual data into a structured dataset, resulting in a collection of approximately 14,532 frames with a total size of 5.36 GB. Each frame represented a snapshot of the frequency spectrum over time, capturing normal, untampered signals for further analysis.

To assess anomaly detection methods, we replicated this entire process using manipulated data to create a second dataset. The manipulation was introduced by altering specific characteristics of the transmitted radio frequency signals to simulate tampered or anomalous behavior. After conducting the same four-hour recording and frame extraction procedure, we obtained a second dataset consisting of approximately 12,139 frames, totaling 3.13 GB in size. This manipulation allowed us to generate realistic abnormal signals for use in anomaly detection experiments, ensuring a robust and comprehensive dataset for training and evaluation of our machine learning models.

### 4.2. System of Tampered Frequency Anomalies Using Machine Learning Techniques

The purpose of the proposed Anomaly Detection system is to analyze both received and broadcast data signals in order to detect anomalies in LoRa networks. The fundamental strategy involves scrutinizing LoRa transmission characteristics to spot deviations that may signal tampering or signal compromise. Our approach integrates multiple machine learning algorithms to improve detection accuracy and effectiveness.

The system is composed of two primary modules: data preprocessing and anomaly detection. The preprocessing module handles the collection and preparation of LoRa transmission data, including formatting and organizing signal data for subsequent analysis. It extracts pertinent features from the data to support effective anomaly detection.

When relevant, the anomaly detection module determines the type of abnormality by applying advanced machine learning models to classify these features as either normal or anomalous. This module uses several methods, including Principal Component Analysis, Autoencoder, Isolation Forest, Local Outlier Factor, and Variational Autoencoder, to identify anomalies in LoRa-based IoT networks in particular.

Overall, the system aims to ensure the security and reliability of LoRa communications by detecting and classifying anomalies. By leveraging these advanced machine learning models, the system strives to effectively identify and address deviations in signal patterns, thereby safeguarding the integrity of LoRa network operations.

#### 4.2.1. Autoencoder-Based Tampered RF Transmission Detection

In this section, we employed the unsupervised machine learning technique of autoencoders and provided a comprehensive explanation of its application. Autoencoders are widely used neural networks in unsupervised learning, especially for purposes such as dimensionality reduction, feature extraction, and detecting anomalies.

Mathematical Formulation

Let $x\in R^{n}$ be the input data. The encoder maps *x* to a latent representation $z\in R^{m}$ using a function f(x), where m<n. The decoder maps *z* back to the original space using a function g(z). The reconstruction error has to be minimized, and this is usually achieved by measuring it with a loss function such mean squared error (MSE):

$$L\left(x,g\left(f\left(x\right)\right)\right)={∥x-g(f(x))∥}^{2}$$

Data preparation and Data Splitting The initial process involves loading and preprocessing the image data from two directories: one for normal images and another for anomalous ones. The ‘load_images’ function processes the images by resizing them to 64 × 64 pixels, normalizing the pixel values to a range between 0 and 1, and converting them into numpy arrays for efficient storage and further analysis.

The normal images are split using the ‘train_test_split’ function into training and validation sets in order to prepare the data for model training. In order to evaluate how well the model performs on fresh, untested normal data, partitioning is necessary to guarantee that the model is trained on one portion of the normal data and validated on a different subset.

Building the Autoencoder Different neural network topologies can be used to build autoencoders. The degree of compression and reconstruction accuracy is determined by the number of neurons in each layer of a typical architecture, which consists of several layers of neurons. While convolutional layers (for image data) or recurrent layers (for sequential data) may be used in more complex architectures, fully connected layers are the most basic type of autoencoder. Since we have image data in our situation, convolutional layers are used.

**Encoder:** The encoder part of the autoencoder reduces the dimensionality of the input data. This is performed through a series of linear transformations followed by nonlinear activations. For example, a basic encoder might use a series of dense (fully connected) layers with ReLU activations:

z=f(x)=σ(Wx+b)

where *W* and *b* are the weights and biases of the layer, and σ is a nonlinear activation function.

**Decoder:** The compressed representation is used by the decoder to reassemble the input data. Typically, this involves utilizing a sequence of thick layers with nonlinear activations in reversal of the encoding process:

$$\hat{x}=g\left(z\right)=\sigma^{\prime }\left(W^{\prime }z+b^{\prime }\right)$$

where $W^{\prime }$ and $b^{\prime }$ are the weights and biases of the decoder layers, and $\sigma^{\prime }$ is a nonlinear activation function.

Training Autoencoder: In order to reduce overfitting and improve training efficiency, the model makes use of early stopping and learning rate adjustment callbacks. While ReduceLROnPlateau adjusts the learning rate in response to changes in the validation loss, early halting ends the training process when the validation loss stops improving.

Anomaly Detection The model is used to rebuild the anomalous and validation images after it has been trained. Anomalies are found using the reconstruction error, which is calculated as the mean squared error between the original and reconstructed pictures. Based on the 90th percentile of reconstruction errors from the validation dataset, a threshold for anomaly identification is set. Reconstruction errors greater than this level are categorized as abnormal images.

#### 4.2.2. LOF-Based Tampered RF Transmission Detection

An unsupervised method for identifying anomalies is the Local Outlier Factor, which measures the density of data points. It excels in finding outliers in complex datasets where density plays a crucial role. According to the local density principle, which underpins LOF, each data point’s anomaly score is determined by how isolated it is from its neighbors. This method works well for identifying local anomalies, or examples that may be common in some regions of the data space but uncommon in others.

By comparing the density surrounding a data point to that of its neighbors, the LOF algorithm calculates the local deviation of a given point. The following steps are involved in computing LOF:

**Compute k-Distance**

For a specific point *p*, the k-distance refers to the distance to its k-th nearest neighbor. This metric is used to identify the local neighborhood surrounding the point.

**Reachability Distance**

The definition of the reachability distance between two points, *p* and *o*, is as follows:

$${\mathrm{reachability}\_\mathrm{distance}}_{k}\left(p,o\right)=\max\left(k-\mathrm{distance}\left(o\right),\mathrm{distance}\left(p,o\right)\right)$$

**Local Reachability Density (LRD)**

The reciprocal of the average reachability distance from a point’s k-nearest neighbors yields the local reachability density of that point, *p*.

$${\mathrm{LRD}}_{k}\left(p\right)={\left(\frac{\sum_{o\in N_{k}\left(p\right)}{\mathrm{reachability}\_\mathrm{distance}}_{k}\left(p,o\right)}{|N_{k}\left(p\right)|}\right)}^{-1}$$

**Local Outlier Factor**

The LOF score for a point *p* is determined by averaging the ratio of *p*’s local reachability density to the local reachability densities of its k-nearest neighbors.

$${\mathrm{LOF}}_{k}\left(p\right)=\frac{\sum_{o\in N_{k}\left(p\right)}\frac{{\mathrm{LRD}}_{k}\left(o\right)}{{\mathrm{LRD}}_{k}\left(p\right)}}{|N_{k}\left(p\right)|}$$

A LOF score near 1 suggests that the point resides in a region with consistent density, indicating it is likely normal, whereas a score substantially higher than 1 signifies that the point is an outlier.

#### 4.2.3. LOF for Image Anomaly Detection

Using LOF for image anomaly detection involves a sequence of processes, such as extracting features, reducing dimensionality, and calculating anomaly scores.

**Feature Extraction**

Since images are high-dimensional, the initial step is to derive significant features from them. This can be accomplished through methods such as:

- **Convolutional Neural Networks (CNNs)**: Utilizing pre-trained models such as VGG16, and ResNet, or designing custom CNNs can help in extracting deep features from images.
- **Traditional Methods**: Techniques like Scale-Invariant Feature Transform (SIFT), Histogram of Oriented Gradients (HOG), or even raw pixel values can be used as features.

**Dimensionality Reduction**

Techniques like Principal Component Analysis (PCA) or t-Distributed Stochastic Neighbor Embedding (t-SNE) are often used to simplify the data and reduce noise because feature vectors from images can be quite high-dimensional.

**Applying LOF**

Once the features are extracted and possibly reduced in dimensionality, the LOF algorithm can be applied:

- **Distance Metric**: Choose an appropriate distance metric (e.g., Euclidean distance) for computing the distances between feature vectors.
- **Selecting k**: The choice of k (number of neighbors) is critical and can be determined through cross-validation or domain knowledge.
- **Anomaly Scoring**: The LOF scores for each image are calculated. Images with LOF scores significantly higher than 1 are considered anomalies.

**Thresholding**

A threshold is established to differentiate normal images from anomalous ones based on LOF scores. This threshold may be set empirically or derived from the LOF score distribution within the training dataset.

#### 4.2.4. Isolation Forest-Based Tampered RF Transmission

Rather than modeling the typical data, the Isolation Forest algorithm concentrates on identifying abnormalities. The foundation of this strategy is the idea that anomalies deviate dramatically from the bulk of data points and are few in number. The system creates several decision trees, or “isolation trees”, in order to isolate these abnormalities. The main finding is that anomalies are isolated faster than regular data points because of their unique characteristics.

In binary trees, each path is made up of a sequence of splits intended to isolate distinct data points. Isolation trees are created by randomly choosing a feature and a split value within that feature’s range at each step. The model’s ability to identify anomalies across several data dimensions is enhanced by the randomness in feature selection and split values, which guarantee variety in the trees.

These isolation trees are utilized in an ensemble within the Isolation Forest. An anomaly score is computed for each data point based on the depth of the leaf node at which isolation occurs. In these trees, anomalies that are isolated and have fewer splits typically have shorter paths. The average path length among all the trees in the forest is used to calculate the anomaly score. Larger anomaly ratings are given to isolated data points at shallow depths, indicating a larger probability that these data points are anomalies.

A data point’s anomaly score is determined by how deeply it can be separated; shorter pathways imply that the point is anomalous since it can be isolated more readily than normal points. The average of the path lengths from every tree makes up the final anomaly score, which offers a reliable indicator of how out of the ordinary a data point is with respect to the dataset.

The Isolation Forest approach divides datasets, including categorical variables, according to category frequencies, using each category as a discrete feature. The distribution of these categories is used to identify anomalies; less frequent or uncommon categories are more likely to be categorized as anomalies.

Application to Image Data Applying the Isolation Forest algorithm to image data involves several steps:

1. **Data Preparation**: Preprocessing is performed on images to normalize pixel values and standardize their size. Transforming high-dimensional picture data into a format suitable for anomaly detection requires this method.
2. **Feature Extraction**: Images are converted into one-dimensional vectors to enable the Isolation Forest algorithm’s application. This conversion allows the algorithm to process the image data as a high-dimensional feature matrix.
3. **Model Training and Evaluation**: A dataset of typical photos is used to train the Isolation Forest model. It is used to find anomalies in validation and anomalous image datasets after training.

**Figure 5:**
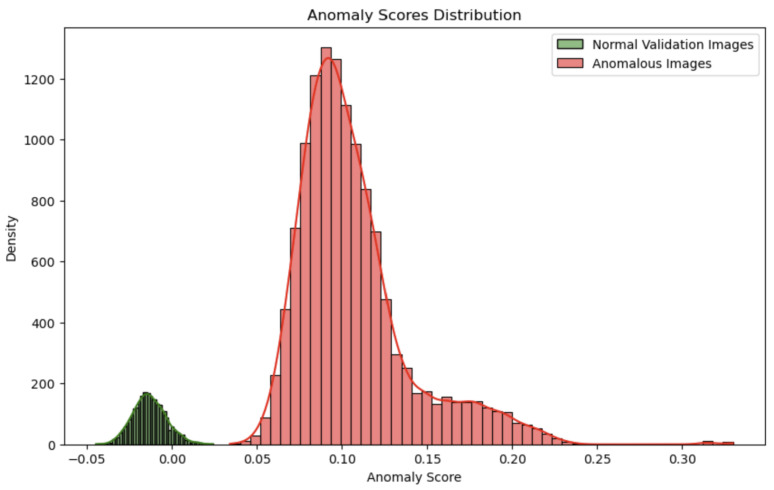
Anomaly scores distribution for isolation forest.

The distribution of anomaly scores generated by the Isolation Forest model for two distinct datasets—the validation set of normal images and the collection of anomalous photos—is shown in Figure 5. The degree to which each image deviates from the typical patterns that the model has learned is shown by the anomaly scores.

In Figure 5, the distribution of scores for the normal validation images is represented in green, while the scores for the anomalous images are shown in red. Ideally, a well-performing model will have the green distribution (normal images) clustered towards lower anomaly scores, demonstrating that these images align closely with the model’s learned definition of normal. In contrast, the red distribution (anomalous images) should be positioned towards higher anomaly scores, signifying that these images are being recognized as outliers. A distinct separation between these two distributions indicates that the model is effectively differentiating between normal and anomalous images. This distinct division would imply that the model has successfully represented the regular data patterns and is capable of identifying patterns that deviate from the norm. Significant overlap between the distributions, however, can indicate that the model is having trouble differentiating between anomalous and normal data, which could result in problems with misclassification.

#### 4.2.5. Principal Component Analysis (PCA)

By utilizing reconstruction errors, Principal Component Analysis (PCA) is a technique used for anomaly detection in image collections. In order to minimize the dimensionality of normal images and capture the most important changes, PCA is trained on them. The PCA model flattens the images, passes them through it, converts them into a lower-dimensional space, and then reconstructs them. To distinguish between normal and anomalous images, reconstruction errors are computed as the mean absolute difference between the original and reconstructed images. To categorize anomalies, a threshold is set at the 95th percentile of the normal errors since normal data are usually reconstructed with smaller mistakes. The image is marked as anomalous if the reconstruction error is greater than this cutoff.

To determine how successfully the model distinguishes between normal and anomalous data, an ROC curve is plotted and the Area Under the Curve (AUC) is computed. Additional metrics, including accuracy, precision, recall, and F1 score, are provided via a confusion matrix and classification report, offering a thorough understanding of the efficacy of the PCA-based anomaly detection model. This method efficiently detects minute variations in picture data using PCA’s dimensionality reduction capabilities, assisting in the identification of anomalies.

#### 4.2.6. Variational Autoencoder (VAE)

A Variational Autoencoder is created especially for visual anomaly detection. The encoder and the decoder are the two main parts of the VAE. In order to sample from a Gaussian distribution in the latent space, the encoder class decreases the dimensionality of the input data and produces two essential values: the mean and log variance. In doing so, the model is able to learn a smooth and continuous latent representation of the input. The decoder class, on the other hand, uses a sigmoid activation function to guarantee that the output values are between 0 and 1 when reconstructing the original input from this latent representation.

Reconstruction loss and Kullback–Leibler (KL) divergence are combined to create a proprietary loss function, which is then used to improve the VAE. Using mean squared error as the metric, the reconstruction loss quantifies how effectively the model replicates the input data, whereas the KL divergence assesses the deviation between the learned latent distribution and a standard normal distribution, promoting a well-organized latent space. The photos are first flattened into 1D arrays and supplied into the model during the training process. Because the VAE is trained only on normal images, anomalies cannot affect its ability to detect underlying patterns.

Reconstruction errors are calculated after training to measure how effectively each image was recreated for both normal and abnormal images. Reconstruction errors of the normal images are used to determine a threshold, and anomalies are defined as images that are above this threshold. The confusion matrix, accuracy, and classification report are some of the metrics used to assess the model’s performance and give information on how well it can distinguish between normal and abnormal data. Lastly, the model’s efficacy in detecting anomalies is evaluated through the generation of visualizations such as the confusion matrix and ROC curve, which demonstrate the VAE’s capability for reliable anomaly identification in picture datasets.

**Difference between Traditional Autoencoder and Variational Autoencoder**

Through the use of the reparameterization trick, which promotes gradient flow during training, the VAE architecture offers a latent space representation in which the encoder outputs both the mean and log variance of a Gaussian distribution. This allows for stochastic sampling. A deterministic mapping, on the other hand, is used by the conventional Autoencoder to produce a single vector that represents the compressed input without taking distributional factors into account. A reconstruction loss and a KL divergence term make up the loss function of the VAE, which encourages the model to learn a structured latent space that approximates a standard normal distribution. In contrast, the traditional Autoencoder only minimizes the reconstruction error, usually with the help of binary cross entropy or mean squared error.

Both models compute reconstruction errors to identify images as normal or anomalous based on a predetermined threshold after training on normal images; however, the VAE outputs also include latent mean and log variance in addition to reconstructed images. The reconstruction error of the VAE is calculated using absolute differences, while the standard Autoencoder utilizes mean squared error, which results in a simpler comparison of anomalies. The VAE is a more potent option for generative tasks because of its probabilistic character, which permits more flexibility and maybe higher performance in anomaly detection tasks, especially in complex datasets, even if both models use similar techniques for visualization and evaluation.

## 5. Experiment and Results

The experimental setup and findings of our work on anomaly detection in LoRa-based IoT networks are presented in this part. The main goal of the experiment was to develop a dataset of both natural and artificial LoRa signals at a frequency of 915 MHz by simulating and analyzing radio frequency transmissions using Software-Defined Radio (SDR) tools, such as GNU Radio and HackRF. Key parameters like bandwidth, BB gain, and intermediate frequency (IF) gain were adjusted to create a variety of signal profiles and record transmissions from MKRWAN 1310 devices in real-time. The dataset was designed to encompass both normal operational signals and anomalies that were intentionally produced through signal alteration or jamming. A range of machine learning methodologies, such as Isolation Forest, Local Outlier Factor (LOF), and Autoencoder, were utilized to assess the effectiveness of anomaly detection. By using evaluation criteria including accuracy, precision, recall, and F1-score, we were able to provide a thorough analysis of each method’s performance and determine which strategies would work best for protecting the security and integrity of LoRa networks. The dataset composition, experimental protocols, and anomaly detection algorithms’ performance outcomes are covered in detail in the ensuing subsections.

### 5.1. Experiment Setup

**Figure 6:**
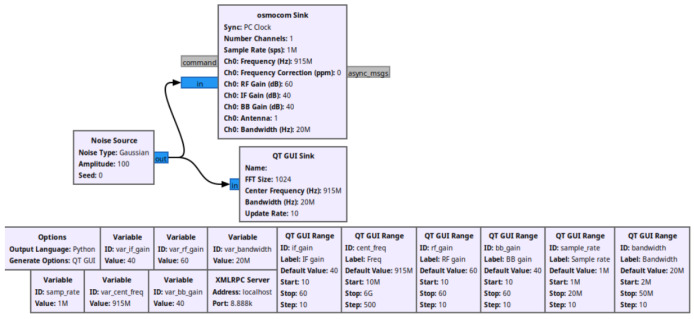
Block diagram of a jamming signal generation flowgraph in GNU Radio.

In this experiment, a Software-Defined Radio (SDR) system was implemented using GNU Radio, leveraging both a Noise Source and an Osmocom Sink to simulate and process radio frequency signals at 915 MHz, a common frequency in LoRa networks. The system used QT GUI Range blocks to allow dynamic control of various parameters, including the intermediate frequency (IF) gain, baseband (BB) gain, sample rate, and bandwidth. The Noise Source generated a Gaussian noise signal, which was passed through the SDR system to simulate random interference in the transmission. The signal parameters were controlled through GUI sliders, with the IF gain and BB gain set at default values of 40 dB, while the sample rate and bandwidth were configured at 1 MHz and 20 MHz, respectively. The center frequency was fixed at 915 MHz to align with LoRa specifications. The osmocom Sink, synchronized with a PC clock, processed the incoming signal and allowed for real-time visualization using a QT GUI Sink. XMLRPC Server functionality was also included for external communication at port 8888. This setup provided a flexible environment for observing signal behavior under different configurations, particularly in relation to SDR-based LoRa transmissions. The GNU Radio structure can be seen in Figure 6, illustrating the setup for generating a Gaussian Noise Source at 915 MHz with adjustable parameters such as sample rate, IF gain, BB gain, RF gain, and bandwidth. The system is configured to send the generated signal to an Osmocom Sink, simulating real-time transmission for interference analysis.

**Figure 7:**
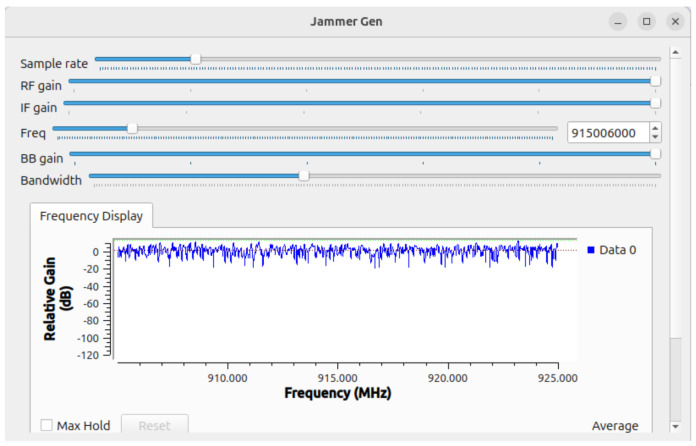
Interface of the jamming signal generator.

Figure 7 displays the graphical interface of a jamming signal generator implemented in GNU Radio. The interface provides controls for adjusting key parameters such as the sample rate, RF gain, IF gain, center frequency, BB gain, and bandwidth. The sliders allow real-time modifications to these parameters, giving users flexibility in controlling the characteristics of the transmitted jamming signal.

The center frequency is set to approximately 915 MHz, which is within the LoRa frequency range, and the graphical plot below shows the relative gain (dB) over a frequency range between 910 MHz and 925 MHz. The plot represents the jamming signal’s effect on the frequency spectrum, where the signal exhibits relatively constant power across the entire bandwidth of interest, creating a noise-like profile that can interfere with legitimate communications operating within this range.

The ability to dynamically adjust parameters such as RF gain, IF gain, and bandwidth in real-time allows us to examine how different signal configurations affect jamming efficiency and signal interference. The “Max Hold” feature, though not selected here, could be used to retain the peak values of the signal over time, enabling the analysis of the maximum interference generated.

The dataset creation involved several key steps:

1. **Signal Acquisition**: We configured the MKRWAN 1310 transmitter to operate at 915 MHz with a bandwidth of 125 kHz. The transmitter sent a series of packets, including both typical and manipulated signals to simulate anomalies.
2. **Data Collection**: One HackRF SDR is used to record the transmitted signals. These recordings were then split into individual frames, resulting in a dataset comprising numerous frames of normal and anomalous data.
3. **Dataset Composition**: We created two separate datasets: one for normal transmission signals and one for manipulated or anomalous signals with others. The normal dataset comprised typical transmission signals reflecting standard operational conditions. The anomalous dataset was designed to include intentional disturbances and alterations to simulate various potential anomalies. These malicious signals were generated by HackRF.
4. **Data Preparation**: The collected frames are processed and added into the classified folder as normal or anomalous. This folder is used to build a comprehensive dataset, enabling detailed analysis and testing of various anomaly detection methods.

This experimental method established a diverse and comprehensive dataset, which served as a strong basis for assessing the effectiveness of various anomaly detection methods in LoRa communications.

### 5.2. Evaluation Metrics

We evaluated the effectiveness of the anomaly detection methods on our dataset using a number of key metrics that provide information about how well these approaches distinguish between normal and aberrant signals. Among the primary metrics employed are:

- **Accuracy**: This measure assesses the proportion of cases—both normal and anomalous—that are accurately identified in relation to the total number of cases. It functions as a broad indicator of the model’s efficacy. One measures accuracy using:
  $$\mathrm{Accuracy}=\frac{\mathrm{Number}\;\mathrm{of}\;\mathrm{Correct}\;\mathrm{Predictions}}{\mathrm{Total}\;\mathrm{Number}\;\mathrm{of}\;\mathrm{Predictions}}$$
- **Precision**: Out of all cases designated as positive (including both true positives and false positives), precision reflects the percentage of true positive predictions (properly recognized aberrant signals). It is calculated as follows and shows the model’s accuracy in identifying positive instances:
  $$\mathrm{Precision}=\frac{\mathrm{True}\;\mathrm{Positives}}{\mathrm{True}\;\mathrm{Positives}+\mathrm{False}\;\mathrm{Positives}}$$
- **Sensitivity**: The recall measures the percentage of actual aberrant signals, or true positive events, that the model is able to identify. It is based on the following and indicates the model’s capacity to find all pertinent anomalies:
  $$\mathrm{Recall}=\frac{\mathrm{True}\;\mathrm{Positives}}{\mathrm{True}\;\mathrm{Positives}+\mathrm{False}\;\mathrm{Negatives}}$$
- **F1-Score**: Through the computation of their harmonic mean, the F1-score integrates recall and precision into a single statistic. Because it offers a balanced measure of recall and precision, this score is especially useful in situations when class distributions are unbalanced. It is calculated with the help of:
  $$F1-\mathrm{Score}=2\times \frac{\mathrm{Precision}\times \mathrm{Recall}}{\mathrm{Precision}+\mathrm{Recall}}$$

When taken as a whole, these metrics offer a comprehensive assessment of the performance of the anomaly detection techniques, guaranteeing that the efficacy and classification accuracy of the methods are appropriately evaluated.

### 5.3. Results and Analysis

**Figure 8:**
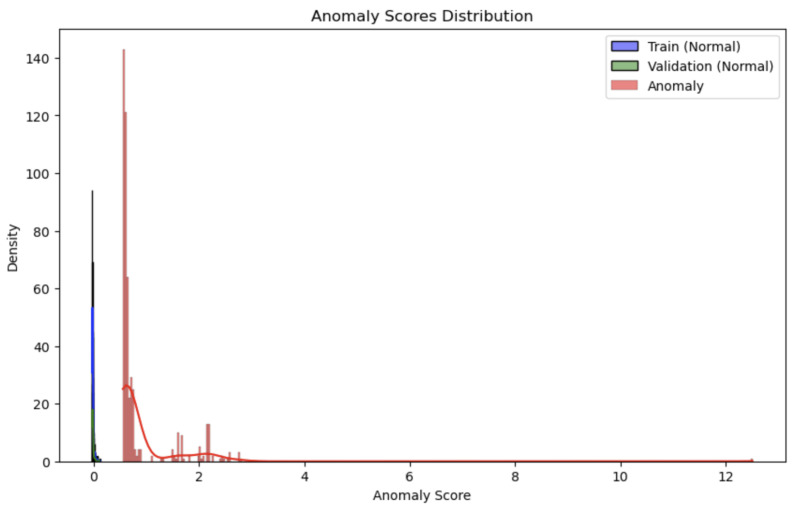
Anomaly scores distribution for LOF.

The LOF model excelled in detecting anomalies, achieving an impressive accuracy of 97.78%, correctly classifying 98% of the images. Its precision of 0.98 signifies it was nearly flawless in identifying anomalies, and a recall of 0.98 shows it detected nearly all true anomalies. With an F1 score of 0.98, the LOF model demonstrates a highly balanced performance. Figure 8 illustrates the anomaly score distribution across three datasets: the training and validation sets of normal images, and the set of anomalous images. **The close clustering of normal images and the spread of anomaly scores reflect the model’s capability to effectively distinguish between normal and anomalous data, with a clear separation in scores.**

The Autoencoder model achieved an accuracy of 97.27%, performing well in classifying normal and abnormal images. With a precision of 0.98, it was correct in its anomaly classifications 98% of the time, reflecting reliable anomaly identification. The recall of 0.98 indicates that it detected 98% of the actual anomalies, demonstrating strong sensitivity but missing some. The F1 score of 0.98 balances precision and recall, highlighting the Autoencoder’s solid overall performance, successfully identifying anomalies while managing the trade-offs between false positives and false negatives.

The Isolation Forest model achieved a strong accuracy of 84.49%, effectively classifying data points as either normal or anomalous. With a precision of 0.88, it correctly identified anomalies in 88% of cases. The recall of 0.84 reflects its ability to detect the most true anomalies, and the F1 score of 0.84 shows that the model balances precision and recall reasonably well, highlighting its overall reliability.

Principal Component Analysis (PCA) also performed admirably, with an accuracy of 97.27%, precision of 0.97, recall of 0.97, and an F1 score of 0.97, showing consistent performance in identifying anomalies with minimal errors.

Similarly, the Variational Autoencoder demonstrated an accuracy of 97.27%, with a precision of 0.97, recall of 0.97, and an F1 score of 0.97, mirroring the performance of the Principal Component Analysis and traditional Autoencoder, indicating a strong ability to differentiate between normal and anomalous data.

**Figure 9:**
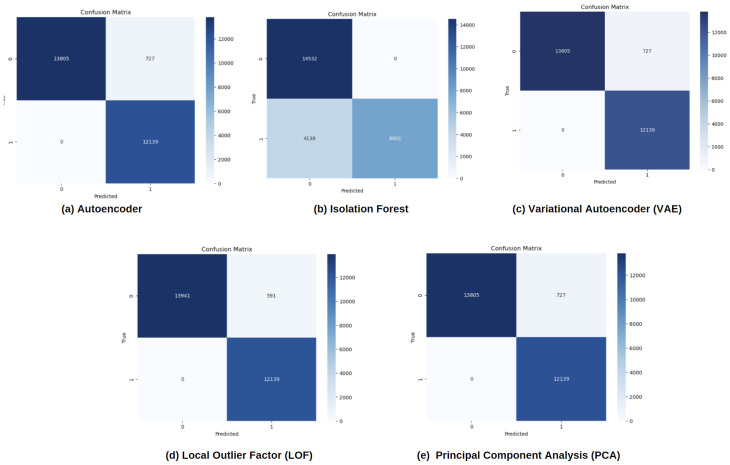
Confusion matrices showing the performance of five anomaly detection algorithms—(**a**) Autoencoder, (**b**) Isolation Forest, (**c**) Variational Autoencoder, (**d**) LOF, and (**e**) Principal Component Analysis (PCA)—in classifying normal and anomalous data. Darker shades represent correct classifications (true positives and true negatives), while lighter shades show misclassifications (false positives and false negatives).

Figure 9 presents confusion matrices for the Autoencoder, Isolation Forest, Variational Autoencoder, LOF, and Principal Component Analysis algorithms. These matrices are a visual representation of how each model performs in terms of correctly classifying normal and anomalous images. Section 5.2 has a detailed explanation of how these matrices are calculated. In these matrices, a well-performing model is characterized by a higher number of True Positives and True Negatives (darker areas), indicating more accurate classifications, and fewer False Positives and False Negatives (lighter areas). Comparing the matrices across the five algorithms shows that models such as LOF and Principal Component Analysis exhibit minimal misclassifications, signifying their strong performance in anomaly detection tasks, while others like the Autoencoder demonstrate more balanced but less precise classifications.

In summary, Table 1 highlights the performance of five anomaly detection algorithms—Autoencoder, Local Outlier Factor (LOF), Isolation Forest, Principal Component Analysis (PCA), and Variational Autoencoder (VAE). The LOF model achieved the highest precision, recall, and F1 score, with values of 0.98 across these metrics, demonstrating its effectiveness in identifying anomalies. Both the Principal Component Analysis and VAE achieved identical scores of 97.27% accuracy, along with precision, recall, and F1 scores of 0.97, indicating strong performance. The Autoencoder also performed well, matching the Principal Component Analysis and VAE in accuracy and achieving a balanced trade-off between precision and recall. In contrast, the Isolation Forest exhibited lower metrics, with an accuracy of 84.49% and precision, recall, and F1 scores of 0.88, 0.84, and 0.84, respectively. This work underscores the significance of advanced anomaly detection methods for securing large-scale IoT environments, particularly in LoRa networks. The application of image-based anomaly detection techniques, including Principal Component Analysis, Autoencoder, and Isolation Forests, enhances the visualization of frequency patterns, effectively revealing subtle tampering or interference within the data. By leveraging these advanced methods, robust solutions for identifying anomalies in complex IoT signal patterns are provided, contributing to improved security and reliability in these systems.

**Figure 10:**
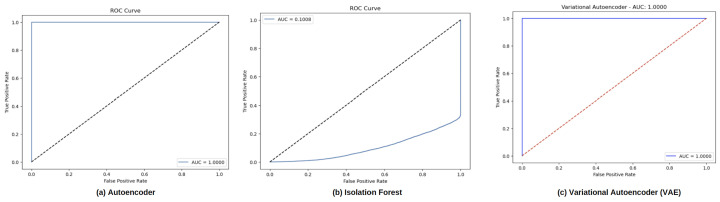
ROC curves for traditional (**a**) autoencoder, (**b**) isolation forest and (**c**) variational autoencoder.

**Figure 11:**
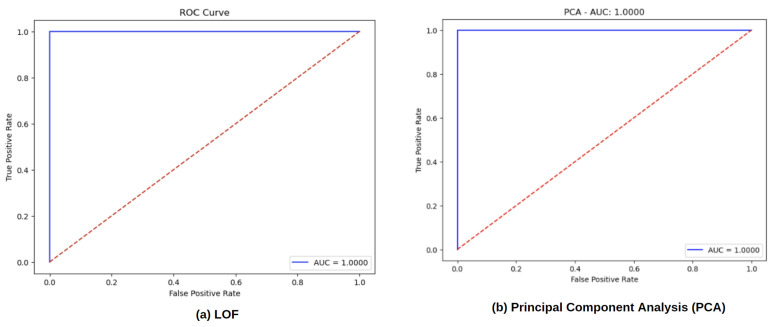
ROC curves for (**a**) LOF and (**b**) Principal Component Analysis (PCA).

Figure 10 depicts the Receiver Operating Characteristic (ROC) curves for five different anomaly detection models: (a) Autoencoder, (b) Isolation Forest, (c) Variational Autoencoder (VAE), and Figure 11 depicts (a) Local Outlier Factor (LOF), and (b) Principal Component Analysis (PCA). Each ROC curve represents the trade-off between the true positive rate (sensitivity) and the false positive rate across various decision thresholds, with the area under the curve (AUC) displayed in each plot. It can be observed that the Autoencoder, Variational Autoencoder, Local Outlier Factor, and Principal Component Analysis models performed well. This indicates that the model was able to accurately differentiate between normal and tampered signals under the test conditions, with nice classification results.

The Isolation Forest model, while highly effective, showed lower performance compared to other methods. Although it was still very accurate, there were instances where it misclassified the data, suggesting that it may not be as precise in identifying anomalies.

**These results suggest that the Autoencoder, Variational Autoencoder (VAE), LOF, and Principal Component Analysis (PCA) models are well-equipped to handle the detection of tampered signals without significant performance issues.** The slightly reduced effectiveness of the Isolation Forest indicates that while still reliable, it may be less robust in this particular environment compared to the other models.

## 6. Conclusions

The Local Outlier Factor (LOF) method performed exceptionally well in our evaluation, achieving the highest accuracy of 97.78%, along with outstanding precision, recall, and an F1 score of 0.98. This highlights its remarkable efficacy in anomaly detection. In addition, the Principal Component Analysis and Variational Autoencoder also demonstrated excellent performance, both achieving an accuracy of 97.27% and maintaining consistent precision and recall values. The Autoencoder mirrored this performance with the same accuracy metrics. However, the Isolation Forest exhibited significantly lower performance, with an accuracy of only 84.49%, indicating challenges in effectively identifying anomalies compared to the other methods.

There are several challenges to note in the current framework. The performance of most algorithms, especially LOF, can depend heavily on the quality and quantity of the training data. Insufficient anomalous data may result in reduced effectiveness. Furthermore, LOF requires careful parameter tuning, and the traditional Autoencoder has high computational demands during training, making it less suitable for real-time anomaly detection, particularly in resource-constrained environments.

To address these limitations, future work should explore adaptive learning mechanisms that enhance the framework’s flexibility and robustness across diverse IoT settings. Additionally, expanding the framework to support multiple IoT communication protocols and optimizing it for real-time anomaly detection will improve its overall applicability. Research into advanced feature engineering, comprehensive benchmarking, and the integration of anomaly detection with broader security systems will further enhance the reliability and versatility of detection systems in LoRa-based IoT networks. In conclusion, this paper highlights the pressing necessity for deploying advanced security measures in embedded systems and also environmental measurements. Integrating machine learning models into an intrusion detection system (IDS) framework provides a robust approach to safeguarding embedded systems from the growing complexity of cyber threats.

## Figures and Tables

**Table 1:** Performance metrics for anomaly detection algorithms.

Algorithm	Accuracy	Precision	Recall	F1 Score
LOF	97.78%	0.98	0.98	0.98
Autoencoder	97.27%	0.97	0.97	0.97
Isolation Forest	84.49%	0.88	0.84	0.84
Principal Component Analysis	97.27%	0.97	0.97	0.97
VAE	97.27%	0.97	0.97	0.97

## Data Availability

The dataset used in this project was generated from a dedicated testbed located in the Autonomous Systems and IoT Lab within the Computer Science Department at Sam Houston State University. All datasets are securely stored in the department’s data center and the lab’s computing system. These datasets are exclusively available to our research team for conducting various experiments.

## Abbreviations

The following abbreviations are used in this manuscript:

IoT	Internet of Things
LOF	Local Outlier Factor
LoRa	Long-Range Modulation
LoRaWAN	Long-Range Wide Area Network
LOFAR	Low-Frequency Array
ROAD	Radio Observatory Anomaly Detector
HPC	High-Performance Computing
PCA	Principal Component Analysis
IDS	Intrusion Detection System
MitM	Man in the Middle
CR-AE	Convolutional Recurrent AutoEncoder
ConvLSTM	Convolutional Long Short-Term Memory
KL	Kullback–Leibler
VAE	Variational Autoencoder
